# Enhancing Acclimatization of Micropropagated *Pistachio* Through Optimization of Light Spectrum and Vapor Pressure Deficit

**DOI:** 10.3390/plants15030460

**Published:** 2026-02-02

**Authors:** Maryam Davarzani, Saeedeh Zarbakhsh, Saadat Sarikhani, Mahmoud Reza Roozban, Saeid Eshghi, Sasan Aliniaeifard, Gniewko Niedbała, Kourosh Vahdati

**Affiliations:** 1 Department of Horticulture, College of Aburaihan, University of Tehran, Tehran 3391653755, Iran; maryamdavarzani@ut.ac.ir (M.D.); mroozban@ut.ac.ir (M.R.R.); aliniaeifard@ut.ac.ir (S.A.); kvahdati@ut.ac.ir (K.V.); 2 Department of Horticultural Science, College of Agriculture, Shiraz University, Shiraz 7194684354, Iran; saeedeh.zarbakhsh@yahoo.com (S.Z.); eshghi@shirazu.ac.ir (S.E.); 3 Controlled Environment Agriculture Center, College of Aburaihan, University of Tehran, Tehran 3391653755, Iran; 4 Department of Biosystems Engineering, Faculty of Environmental and Mechanical Engineering, Poznań University of Life Sciences, Wojska Polskiego 50, 60-627 Poznań, Poland

**Keywords:** acclimatization, *Pistacia* spp., photosynthetic pigments, red–blue LED

## Abstract

The light spectrum and vapor pressure deficit (VPD) are key environmental factors that significantly influence the morphophysiological development and survival of micropropagated woody plants during acclimatization. However, few studies have focused on their interactive effects under ex vitro conditions. This study examined the combined effects of four light spectra (white, blue, red, and red–blue) and two VPD levels (low: 0.2 kPa; high: 1.0 kPa) on growth, photosynthesis pigments, biochemical indices, and leaf temperature of *Pistacia* spp. ‘UCB1’ plantlets over a 30-day acclimatization period. The results demonstrated that red–blue light under low VPD significantly enhanced plantlet performance across multiple parameters, resulting in the highest leaflet number (79.25 pieces), stem diameter (2.13 mm), leaf dry weight (0.048 g), leaf fresh weight (0.15 g), root length (1.48 cm), and leaf area (103.3 cm^2^). Furthermore, this treatment markedly increased anthocyanin, total soluble carbohydrate content, and photosynthetic pigments (chlorophyll a, chlorophyll b, and carotenoids). Principal component and correlation analyses identified that red–blue light under low VPD was strongly associated with traits linked to growth and photosynthetic ability, whereas blue and white light under high VPD showed the weakest responses. Entropy-weighted TOPSIS ranked red–blue light under low VPD as the most effective treatment for balanced morpho-physiological functions during acclimatization. These findings highlight the importance of optimizing spectral quality and VPD to enhance autotrophic transition and ex vitro establishment in pistachio plantlets. These findings are important for improving ex vitro survival and large-scale propagation efficiency of micropropagated pistachio plantlets.

## 1. Introduction

A considerable proportion of micropropagated plants fail to survive the transition from laboratory conditions to greenhouse or field environments [1]. Acclimating plants to external conditions is often the most challenging step in in vitro propagation protocols and remains a key bottleneck in the micropropagation of many species [1,2]. Physiological disorders causing the death of micropropagated plants are primarily due to excessive water loss resulting from impaired stomatal function, absence of leaf surface wax, thin epidermis, and dysfunctional root systems [3,4].

The propagation of woody plant species in vitro poses greater challenges than that of herbaceous species [5]. The acclimatization process of tissue-cultured plants is significantly influenced by in vitro environmental conditions, including light quality, which affects photosynthetic efficiency and stomatal development [6].

A wide range of endogenous and environmental stimuli stimulate a sophisticated network of signaling pathways that regulate ion channels and solute transporters to control stomatal movements [7]. Under optimal circumstances, this regulatory system is remarkably robust. However, specific environmental deviations—particularly those encountered in controlled environments—can impair its function, preventing stomatal closure in response to stimuli that typically induce this response [8]. Notably, such disruption has been reported in plants cultivated in vitro, particularly under low vapor pressure deficit (VPD) conditions [9]. VPD, defined as the difference between saturated and actual air vapor pressures, acts as a key indicator of the evaporative demand placed on plant tissues [10]. Low VPD reduces transpiration rates and alters stomatal behavior, often leading to dysfunctional stomata that fail to respond to critical closure signals such as darkness, abscisic acid (ABA), and elevated intracellular calcium (Ca^2+^) concentrations [11]. During acclimatization, micropropagated plants may experience mortality due to abiotic and biotic stresses in the external environment, such as elevated temperatures, intense light, low relative humidity, and soil-borne microorganisms and pathogens. These plants often exhibit alterations in morphological, anatomical, or biochemical traits, which need to be adjusted during acclimatization to enhance their adaptation to ex vitro conditions [5].

The detrimental impact of elevated air humidity (low VPD < 0.4 kPa) on stomatal function becomes especially apparent when micropropagated plantlets are exposed to ambient air. High humidity during in vitro development reduces transpiration and impairs cuticle and wax layer formation, resulting in non-functional stomata [12]. Careful adjustment of VPD during ex vitro acclimatization helps maintain optimal stomatal function, enhancing photosynthetic efficiency and ultimately improving plant survival and growth [13].

Adjustments in environmental parameters—including light quality, air temperature, humidity [14], and CO_2_ concentration [9]—have been proposed as strategies to enhance photosynthetic competence during culture. Among these factors, VPD and light spectra have particularly strong influences on stomatal physiology and water regulation. Light quality, particularly blue light, not only regulates stomatal aperture but also interacts with VPD to modulate transpiration, water balance, and morphological development during ex vitro acclimatization [15].

Light quality has a profound effect on stomatal regulation and growth physiology of in vitro-cultured seedlings [16]. Blue light directly affects stomatal guard cells through specific photoreceptors and stimulates stomatal opening [17]. Several studies have demonstrated that plants grown under LED lighting exhibit superior growth, reduced water loss, and higher survival rates during acclimation compared to plants grown under FL light [18]. Interactions between red and blue photoreceptors improve developmental outcomes and support balanced shoot and root development [19,20]. White LEDs, encompassing a broader spectrum, have shown positive effects on organogenesis and antioxidant capacity in in vitro cultures [21].

Despite increasing interest in spectral modulation, strategies to integrate VPD control with light spectrum adjustments have not yet been fully elucidated, especially in woody micropropagated species of high economic importance, such as pistachio. Most studies have either focused on single factors or on herbaceous crops [22], leaving a gap in optimizing combined environmental regimes for woody trees. Moreover, ex vitro transfer is often associated with severe water stress due to immature root systems and non-functional stomata, challenges intensified under low ambient humidity [23,24]. Some approaches, such as gradual hardening, application of antitranspirants such as ABA, and CO_2_ enrichment, have shown promise in facilitating acclimatization [25,26]. However, systematic evaluation of light–humidity interactions during acclimatization of woody species remains limited. Therefore, the present study investigates the interactive effects of VPD and light spectral quality on morpho-physiological attributes of pistachio plantlets under ex vitro conditions. By integrating multivariate analyses, including principal component analysis (PCA), correlation matrices, and entropy-weighted Technique for Order of Preference by Similarity to Ideal Solution (TOPSIS), the study identified optimal sets of treatments that increase photosynthesis, support balanced shoot and root development, and improve acclimatization efficiency.

## 2. Results

### 2.1. Growth-Related Traits and Leaf Temperature

**Figure 1:**
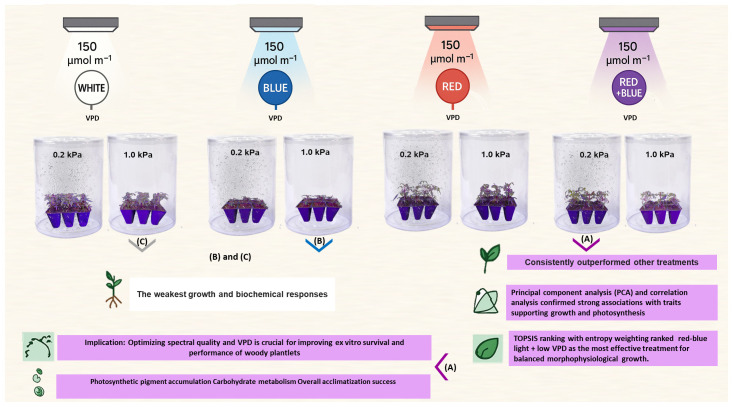
Representative images of ‘UCB1’ pistachio plantlets grown under combined light quality, including RB (red–blue), R (red), B (blue), W (white), and VPD 0.2 (low) and 1.0 (high) kPa treatments. (A) RB light treatment combined with low VPD; (B) B light treatment combined with high VPD; and (C) W light treatment combined with high VPD.

**Figure 2:**
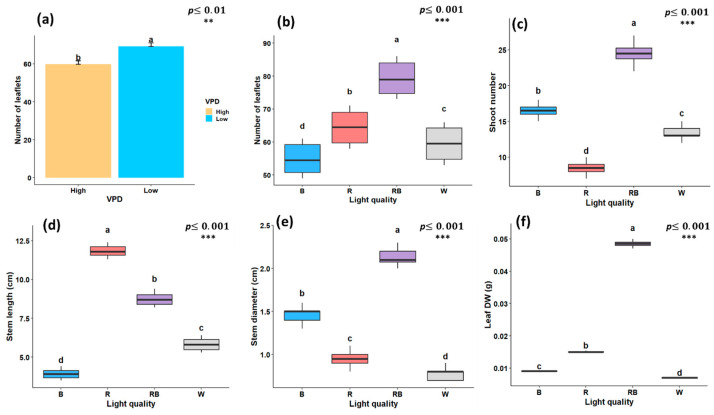
Growth traits of in vitro grown pistachio plantlets under different light spectra and vapor pressure deficit (VPD) conditions in controlled laboratory environment, (**a**) number of leaflets under two VPD levels; (**b**) number of leaflets; (**c**) number of shoots; (**d**) stem length; (**e**) stem diameter; and (**f**) leaf dry weight (Leaf DW) under different light quality treatments. RB, red-blue; R, red; B, blue; W, white; Low, low vapor pressure deficit; High, high vapor pressure deficit. Each treatment consisted of six biological replicates. Statistical significance at the 0.01 probability level is indicated by **, and significance at the 0.001 probability level is indicated by ***. In each figure, letters (a to d) denote statistically significant groupings; treatments sharing at least one letter are not significantly different at the 0.001 probability level.

**Figure 3:**
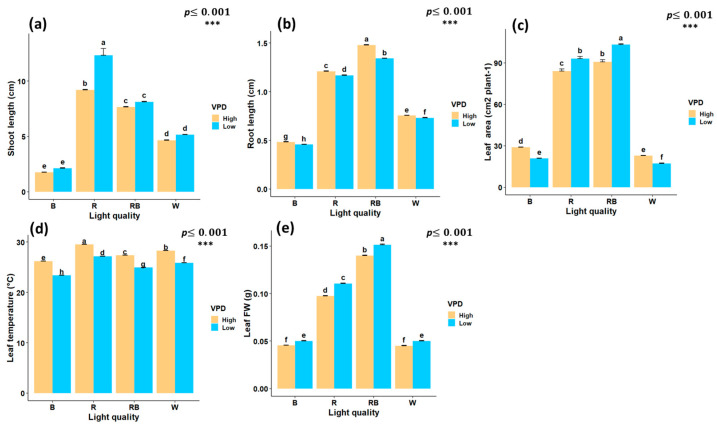
Interaction effects of light quality and vapor pressure deficit (VPD) treatments on growth traits of in vitro grown pistachio plantlets, (**a**) shoot length; (**b**) root length; (**c**) leaf area; (**d**) leaf temperature, and (**e**) leaf fresh weight (Leaf FW) in controlled laboratory environment. RB, red-blue; R, red; B, blue; W, white; Low, low vapor pressure deficit; High, high vapor pressure deficit. Each treatment consisted of six biological replicates. Statistical significance at the 0.001 probability level is indicated by ***. In each figure, letters denote statistically significant groupings; treatments sharing at least one letter are not significantly different at the 0.001 probability level.

As illustrated in Figure 1, Figure 2 and Figure 3, the growth characteristics of pistachio plants were significantly affected by light quality and different VPD levels. According to the factorial analysis of variance (ANOVA), light spectral treatment and VPD independently exerted a significant effect (*p* < 0.001) on leaflet number. Among light-treated plants, the maximum number of leaflets was observed under red–blue light (79.25), while the lowest number was recorded under blue light (54.83) (Figure 2a). Furthermore, plantlets grown under low-VPD conditions produced higher leaflet number (69.2) compared to those under high VPD (59.75) (Figure 2b). Shoot number was also significantly influenced by the light spectrum; the highest value (24.5) was observed under red–blue treatment, and the lowest (8.5) under red light (Figure 2c). Regarding stem length, red light promoted the maximum value of stem length, which was 11.8 cm, whereas blue light treatment resulted in the shortest (3.9 cm) (Figure 2d). In contrast, red–blue treatment resulted in a larger stem diameter (2.13 mm) compared to other light treatments, with the smallest stem diameter recorded under white light (0.78 mm) (Figure 2e). when considering only the light treatment, leaf DW was significantly affected, with the highest DW under red–blue treatment (0.048 g) and the lowest under white light (0.007 g) (Figure 2f). 

Red light treatment significantly increased shoot length, with an increase of 12.3 cm under low VPD. Conversely, the lowest shoot length was observed under blue light treatment at both low VPD (2.11 cm) and high VPD (1.75 cm) (Figure 3a). Red–blue light treatment under high VPD produced the longest roots (1.48 cm), whereas blue light under low VPD resulted in the shortest (0.45 cm) (Figure 3b). Leaf area reached its maximum under red–blue light at low VPD (103.3 cm^2^), while the lowest values were observed under blue light at low VPD (20.9 cm^2^) and white light at high VPD (22.8 cm^2^) (Figure 3c).

Leaf temperature was significantly influenced by both light spectrum and VPD. Statistical results for leaf temperature displayed highly significant differences under red light treatment and high VPD (29.5 °C), while the lowest was recorded under blue light treatment with low VPD (23.3 °C) (Figure 3d). The interaction between the light spectrum and VPD significantly influenced leaf FW. The highest leaf FW was obtained under red-blue treatment with low VPD (0.15 g), while the lowest values were recorded under white and high VPD (0.045 g) and blue light with high VPD (0.0455 g) (Figure 3e).

### 2.2. Photosynthetic Pigments and Biochemical Traits

**Figure 4:**
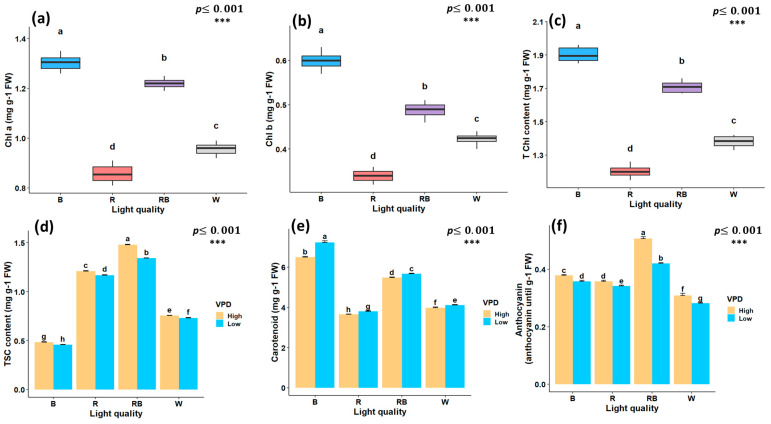
Photosynthetic pigments and biochemical traits of in vitro grown Pistacia plantlets under controlled laboratory conditions. Chlorophyll a (a), chlorophyll b (b), and total chlorophyll (c) were evaluated under different light quality treatments, whereas total soluble carbohydrate content (d), carotenoids (e), and anthocyanins (f) showed significant interaction effects between vapor pressure deficit (VPD) and light quality treatments. TSC, total soluble carbohydrates; Chl a, chlorophyll a; Chl b, chlorophyll b; T Chl, total chlorophyll content. RB, red–blue; R, red; B, blue; W, white; Low, low vapor pressure deficit; High, high vapor pressure deficit. Each treatment consisted of six biological replicates. Statistical significance at the 0.001 probability level is indicated by ***. In each figure, letters denote statistically significant groupings; treatments sharing at least one letter are not significantly different at the 0.001 probability level.

The light spectrum alone had a significant effect on chlorophyll a (Chl a), chlorophyll b (Chl b), and total chlorophyll (T Chl) content. However, the interaction between vapor pressure conditions and light spectrum treatments was not significant. The highest Chl concentrations were observed under blue light, whereas the lowest values were observed under red light treatment (Figure 4a–c). Total soluble carbohydrate content (TSC) was markedly influenced by the interaction between light spectrum and VPD; the highest content was observed under red–blue light treatment at high VPD (1.48 mg/g) and the lowest under blue light at low VPD (0.45 mg/g) (Figure 4d). The carotenoid concentration was highest under blue light treatment with low VPD (7.23 mg/g FW) and was lowest under red light treatment with high VPD (3.65 mg/g) (Figure 4e). Anthocyanin content followed a similar pattern, with the maximum observed under red–blue light at high VPD (0.5 mg/g FW) and the minimum under white light treatment at low VPD (0.28 mg/g FW) (Figure 4f).

### 2.3. Principal Component Analyses (PCA) and Correlation

**Figure 5:**
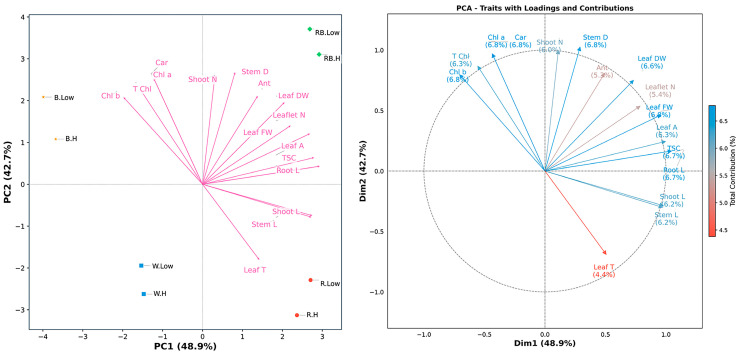
Principal component analysis (PCA-biplot), and contribution of traits along the first two principal components (PC1 and PC2) in the principal component analysis (PCA). Stem L; stem length, Stem D; stem diameter, Shoot L; shoot length, Root L; root length, Shoot N; number of shoots, Leaflet N; number of Leaflets, Leaf DW; leaf dry weight, Leaf FW; leaf fresh weight, Leaf T; leaf temperature, Leaf A; leaf area, Car; carotenoid, TSC; total soluble carbohydrate, Chl a; chlorophyll a, Chl b; chlorophyll b, T Chl; total chlorophyll, Ant; anthocyanin. RB, red–blue; R, red; B, blue; W, white; Low, low vapor pressure deficit; H, high vapor pressure deficit.

To explore the underlying structure and multivariate associations among the measured traits across treatments, a PCA was performed (Figure 5). The first two principal components (PC1 and PC2) explained over 91% of the total variance—48.9% and 42.7%, respectively—under different spectral light and VPD. The first principal component (PC1) was predominantly associated with plant size parameters, which showed the strongest positive correlations from stem length (0.97, 10.56% contribution), shoot length (0.98, 10.73%), and TSC (1.05, 12.24%). Leaf traits, including fresh weight (0.96, 10.28%) and area (1.00, 11.10%), also contributed substantially to this axis. In contrast, all chlorophyll-related traits exhibited negative correlations on PC1, with Chl b (−0.71, 5.57%) showing the strongest inverse relationship. PC2 revealed a distinct photosynthetic pigment axis, with Chl a (0.97, 12.06%) and carotenoids (0.97, 12.06%) demonstrating the highest positive correlations. Stem diameter (1.02, 13.47%) and shoot number (1.00, 12.76%) showed equally strong associations with this axis, while leaf temperature (−0.69, 6.11%) was negatively correlated. The total contribution percentages across both dimensions were remarkably balanced for most traits, ranging from 5.35% (anthocyanin) to 6.78% (stem diameter), with photosynthetic pigments and structural traits showing particularly stable contributions. Notably, total soluble carbohydrates showed exceptional correlation specificity, contributing 12.24% to PC1 but only 0.35% to PC2, indicating this metabolic parameter varies independently from the photosynthetic–structural axis. Table S1 provides comprehensive results for PC1 and PC2.

The PCA biplot indicated that light spectral and VPD treatments were clearly separated along the first two PCs. However, red–blue light under both low and high VPD occupied adjacent positions in the ordination space, reflecting highly similar trait expression profiles despite differences in humidity conditions. This proximity suggests that the positive influence of red–blue light on growth-related parameters, including stem length, shoot biomass, and leaf area, was largely unaffected by VPD variation. In contrast, treatments involving blue or red light, as well as white light, displayed greater spatial separation between low- and high-VPD conditions, which indicates a stronger interaction between the light spectrum and evaporative demand in regulating physiological responses (Figure 5).

**Figure 6:**
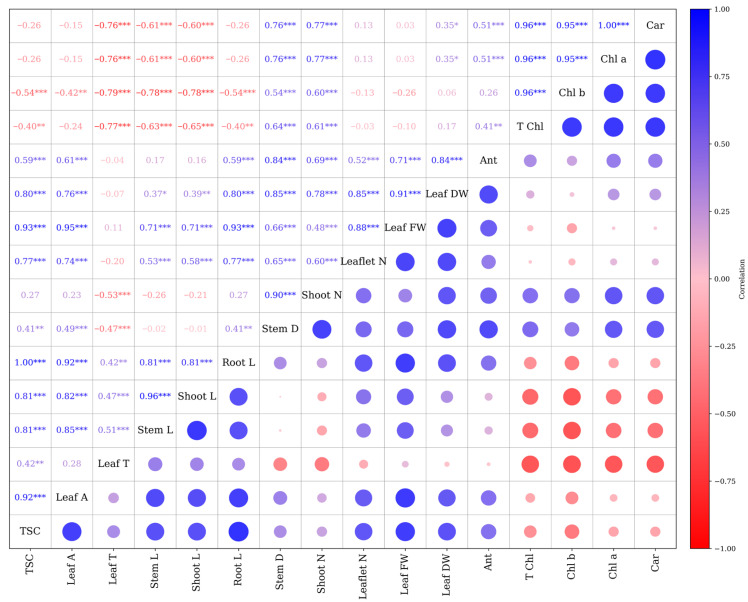
Pearson correlation matrix. Pearson correlation coefficients (* *p* < 0.05, ** *p* < 0.01, *** *p* < 0.001). Stem L; stem length, Stem D; stem diameter, Shoot L; shoot length, Root L; root length, Shoot N; number of shoots, Leaflet N; number of Leaflets, Leaf DW; leaf dry weight, Leaf FW; leaf fresh weight, Leaf T; leaf temperature, Leaf A; leaf area, Car; carotenoid, TSC; total soluble carbohydrate, Chl a; chlorophyll a, Chl b; chlorophyll b, T Chl; total chlorophyll, Ant; anthocyanin.

The correlation analysis revealed several strong and significant associations among the measured morpho-physiological traits (Figure 6). Leaf area showed a strong positive correlation with TSC (r = 0.92 ***), stem length (r = 0.85 ***), and shoot length (r = 0.82 ***). Shoot length exhibited a nearly perfect correlation with stem length (r = 0.96 ***) and a strong positive correlation with root length (r = 0.81 ***). Photosynthetic pigment traits, including Chl a, Chl b, T Chl, and carotenoids, were highly interrelated, with correlation coefficients exceeding r = 0.95 ***. A significant positive correlation was identified between Chl b and TSC (r = 0.96 ***). Conversely, anthocyanin content exhibited weak correlations with most traits, except for a moderate association with Leaf DW (r = 0.41 ***). Negative correlations were observed between photosynthetic pigment traits and several growth parameters, such as root and shoot length. In contrast, traits like leaf temperature exhibited weak to moderate positive associations with vegetative growth. Moreover, the results indicate that leaf area, TSC, and shoot–stem growth are tightly linked, whereas photosynthetic pigment traits form a distinct, highly intercorrelated group, in some cases inversely related to growth variables (Figure 6).

### 2.4. Heatmap and Cluster Analysis

**Figure 7:**
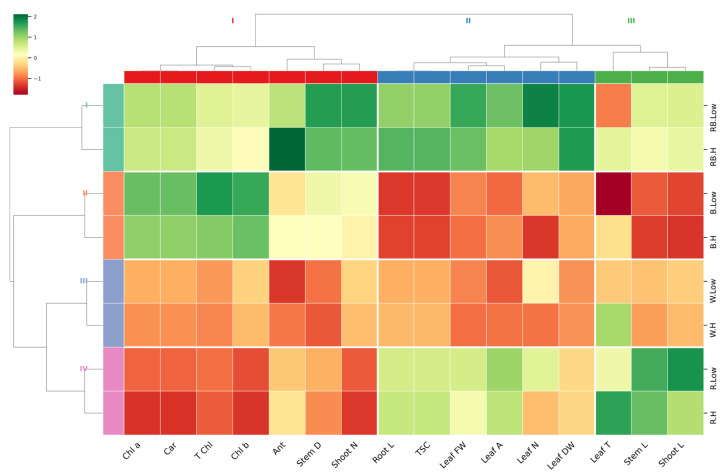
Heatmap with Euclidean distance-based hierarchical clustering on growth and physiological traits. Stem L; stem length, Stem D; stem diameter, Shoot L; shoot length, Root L; root length, Shoot N; number of shoots, Leaflet N; number of Leaflets, Leaf DW; leaf dry weight, Leaf FW; leaf fresh weight, Leaf T; leaf temperature, Leaf A; leaf area, Car; carotenoid, TSC; total soluble carbohydrate, Chl a; chlorophyll a, Chl b; chlorophyll b, T Chl; total chlorophyll, Ant; anthocyanin.

A hierarchical clustering heatmap was performed on the entire experimental data set to visualize the relationship patterns between measured traits under different light and vapor pressure conditions (Figure 7). The hierarchical cluster analysis distinctly clustered treatments based on light quality. Most of the measured parameters showed the lowest correlation in order in white > blue > red, compared to red–blue light under both low and high VPD. However, traits including leaf DW and FW, leaflet number, leaf area, TSC, root length, shoot number, stem diameter, and anthocyanin showed the most significant changes, with high correlations observed under the red–blue light treatment. The dendrogram related to the measured parameters revealed three main clusters: (1) shoot length, stem length, and leaf temperature; (2) leaf DW and FW, leaflet number, leaf area, TSC, and root length; and (3) shoot number, stem diameter, anthocyanin, and photosynthesis pigments. This clustering highlights the distinct physiological and morphological responses of pistachio plantlets to the experimental variables.

### 2.5. Multi-Criteria Treatment Evaluation

**Figure 8:**
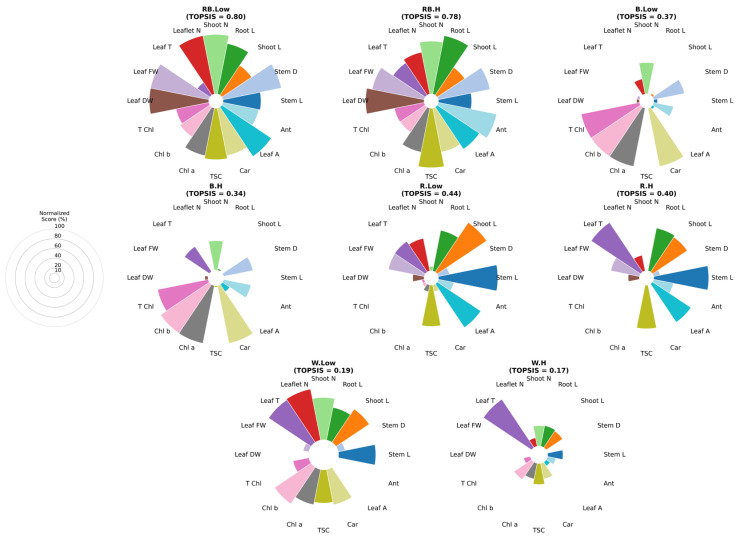
Multitarget analysis based on Entropy–TOPSIS charts of different combinations of light and vapor pressure treatments. Each sector represents one trait, with radius proportional to its Entropy–TOPSIS score. The guide indicates scale levels (20–100%). Stem L; stem length, stem diameter; Stem D, Shoot L; shoot length, Root L; root length, Shoot N; number of shoots, Leaf N; number of leaflet, Leaf FW; leaf fresh weight, Leaf T; leaf temperature, Leaf DW; leaf dry weight, Leaf A; leaf area, Car; carotenoid, TSC; total soluble carbohydrate, Chl a; chlorophyll a, Chl b; chlorophyll b, T Chl; total chlorophyll, Ant; anthocyanin. RB: red–blue, R: red, B: blue, W: white; Low = low vapor pressure, H = high vapor pressure.

The entropy-weighted TOPSIS analysis revealed distinct variations in the overall performance of different light spectra and VPD conditions (Figure 8). Under VPD, the combined red–blue light achieved the highest composite score, outperforming all other treatments, primarily due to superior growth parameters such as stem length, root length, leaf area, and T Chl content. This was followed closely by white light under low VPD, which also showed strong performance across multiple growth and biochemical traits. In contrast, blue light treatments under both low and high VPD exhibited markedly lower rankings, reflecting limited stimulation of both growth and photosynthetic pigment contents, particularly under high-VPD conditions. Red light treatments displayed moderate performance, as red light under low VPD ranked higher than red light under high VPD, indicating that elevated VPD reduced the efficiency of red light in promoting overall plant performance. Interestingly, the combination of red–blue light under high VPD maintained relatively high composite scores, indicating that the effect of red and blue wavelengths partially mitigated the stress induced by higher evaporative demand. Overall, these results highlight the superior adaptability of mixed-spectrum lighting, especially red–blue light under low VPD, in supporting balanced growth development and physiological function under controlled acclimatization conditions.

## 3. Discussion

Micropropagation has emerged as an effective pathway for the large-scale propagation of the commercial pistachio rootstock ‘UCB1’. However, ex vitro acclimatization remains the principal obstacle restricting survival and growth consistency [5]. The transition from the protected in vitro environment to ex vitro conditions exposes plantlets to substantial physiological stress, including impaired photosynthetic activity, limited stomatal control, and altered carbon metabolism [25]. Achieving successful ex vitro acclimatization in woody plants requires the simultaneous regulation of morphogenesis, photosynthetic capacity, and stomatal–cuticular adaptation. Among the environmental parameters available, VPD and light spectral quality play dominant roles. Light and moisture conditions can lead to stomatal dysfunction, a significant reduction in epicuticular wax, and decreased photosynthetic efficiency in micropropagated plants, ultimately reducing acclimation success [27]. LED lights with different spectral compositions have significant effects on micropropagation, but their application in the acclimatization of cultivated seedlings has been less extensively studied [28]. The integration of red and blue light has been shown to enhance morphogenic stability and photosynthetic efficiency [29], particularly under varying VPD conditions. Under low VPD, red–blue light promotes robust growth traits and mechanically stable shoots, which are essential for reducing transplant shock and ensuring long-term field establishment. Although VPD significantly affects plant physiology and productivity, increases in VPD are usually accompanied by concomitant changes in other environmental conditions [30]. However, at elevated VPD, stomatal responsiveness can become impaired, leading to water stress and compromised rooting performance. This highlights the importance of balancing evaporative demand with light cues to maintain functional stomatal activity during the hardening phase [31,32]. ANOVA-based analysis confirmed that red–blue light under low VPD significantly affected growth traits: expanded leaf area, higher leaf biomass, thicker stems, and moderate shoot elongation. This represented an advantageous balance between photosynthetic capacity and ideal phenotype, while maintaining root growth proportional to shoot demand and ensuring an effective source–sink relationship for ex vitro transfer. These coordinated outcomes are consistent with the complementary mechanisms by which blue light triggers stomatal opening via the phototropin–H^+^-ATPase pathway [17], and red light enhances mesophyll photosynthesis and phytochrome-mediated morphogenesis [33].

The results of this study reflect a distinct resource allocation strategy dictated by spectral quality: under certain spectra (like blue), the plant invests heavily in pigment density but limits elongation. Conversely, under others (like red), the plant prioritizes elongation, a characteristic shade avoidance response, at the expense of pigment concentration. This interaction is further illustrated by photosynthetic pigments; while blue-enriched spectra increased chlorophyll and carotenoid concentrations, enhancing both photosynthetic and photoprotective capacity, red light promoted maximal stem length. High-VPD conditions reduce stomatal conductance and photosynthesis while simultaneously increasing plant water loss through transpiration, thereby diverting resources toward stress acclimation [34]. The findings of our study suggest that low VPD conditions may sustain transpirational flux and water balance, enabling the full exploitation of red–blue-induced photosynthetic gains. Photosynthetic pigments further illustrated this interaction: blue-enriched spectra increased chlorophyll and carotenoid concentrations, enhancing both photosynthetic pigments and photoprotective capacity. Under higher VPD, anthocyanin accumulation was stimulated, especially under spectra that supported vigorous growth, reflecting a shift toward antioxidant and membrane-stabilizing strategies [35].

Multivariate analysis provided additional insights, with PC and correlation structures separating a growth index (leaf expansion, biomass, stem/shoot development) and TSC from pigment indexes, including chlorophylls, carotenoids, and anthocyanin contents, which reveal an interaction between structural expansion and pigment accumulation. We interpret that red–blue at low VPD occupied a high-performance niche, combining extensive canopy development and efficient carbon economy with sufficient photoprotection. There have been multiple reports indicating that red–blue spectra optimize leaf anatomy and function over time [36]. Furthermore, implementing red–blue illumination under low VPD during the late in vitro–early ex vitro condition can increase transplantable biomass with stronger shoots, sustain water balance via efficient stomatal control, and prime antioxidant defenses—mitigating key bottlenecks in pistachio micropropagation such as shoot-tip necrosis and hyperhydricity-related fragility.

The current findings highlight a robust effect between red–blue light spectra and VPD for optimizing pistachio ‘UCB1’ rootstock development. While these results point to a coordinated plant response, the specific nature of the interaction between light quality and VPD—whether a direct mechanistic link or an indirect consequence of altered physiology—requires deeper investigation. Subsequent research should prioritize direct physiological validation through comprehensive gas exchange measurements, including net photosynthesis, stomatal conductance, intercellular CO_2_ concentration, and transpiration, paired with chlorophyll fluorescence analysis. This must be integrated with detailed quantification of stomatal density and kinetics to test the hypothesis of enhanced mesophyll–stomatal coordination under red–blue light at low VPD. Stomata are indispensable regulators of plant–atmosphere gas and water exchange, and their activity is a critical indicator of environmental adaptation [37]. Furthermore, molecular analyses focusing on key light-signaling pathways, specifically phototropins, cryptochromes, and phytochromes, as well as abscisic acid-related pathways involving dehydration-responsive element-binding and ABRE-binding factors, are needed to elucidate how spectral and evaporative signals are integrated at the transcriptional level. Ultimately, translating these optimized conditions into phased acclimatization protocols will be essential to ensure that in vitro improvements reliably enhance ex vitro performance and field establishment.

## 4. Materials and Methods

### 4.1. Plant Materials, Experimental Design, and Treatments

Plant material consisted of pistachio rootstock plantlets (‘UCB1’) transferred from in vitro culture to 0.06 L pots filled with a cocopeat–perlite mixture (3:1, *v*/*v*). At the time of transfer, plantlets averaged 2–3 cm in height. The experiment was conducted in 2024 under controlled environmental conditions at the Faculty of Agricultural Technology, University of Tehran, Iran, and kept for 30 days. After transfer to pots, the plantlets entered a pre-acclimatization phase. All plantlets were maintained in a common growth environment with an approximately constant air temperature of 25 °C, and relative humidity inside the packs was regularly monitored using a hygrometer. The low VPD treatment (0.2 kPa) corresponded to a relative humidity of approximately 94%, whereas the high VPD treatment (1.0 kPa) corresponded to a relative humidity of approximately 69%.

During the first 10 days after transfer, all plantlets were maintained under white light at a photosynthetic photon flux density (PPFD) of 150 µmol m^−2^ s^−1^ to minimize transplant shock, and the packs were kept tightly covered to maintain high humidity. The packs were opened every 2–3 days for approximately 5 min for visual inspection and irrigation or nutrient solution application. Thereafter, two VPD levels were gradually imposed over a period of approximately 10 days: half of the plantlets remained under low VPD (0.2 kPa), while the rest were transitioned to high VPD (1.0 kPa) to avoid sudden transpiration stress. Following acclimatization, light treatments including white light (400–700 nm), blue light (460 nm), red light (660 nm), and a red–blue combination (70:30) were applied at a constant PPFD (150 µmol m^−2^ s^−1^). The experiment was arranged as a factorial design with two VPD levels and four light treatments, with six replicates per VPD level within each light treatment (a total of 48 plantlets). The schematic diagram of the experiment is illustrated in Figure 1.

#### 4.1.1. Growth-Related Traits

On the 30th day of light and VPD treatments, since the leaves of in vitro-grown plantlets tend to wilt rapidly upon removal from in vitro culture, plantlets were carefully separated into aerial parts (leaves and stems) and roots immediately after transfer. Growth traits were measured, including stem length (cm), shoot length (cm), and leaf area (cm^2^), determined by analyzing digital images using Digimizer software (v.5.4.9), stem diameter (mm) using a digital caliper, root length (cm), and number of leaflets. Each measurement was conducted on six biological replicates per treatment. Growth parameters such as leaf fresh weight and dry weight (FW and DW; g) were measured with a digital scale (accuracy ± 0.1 mg). For DW measurement, the samples were dried in an forced-air oven at 70 °C for 48 h.

#### 4.1.2. Leaf Temperature

Leaf temperature for each plant was measured using an infrared thermometer (Infrared thermometer, 774 grip Laser, China). To enhance accuracy, six distinct leaves were measured, and the mean of these readings was used for all subsequent analyses. Measurements were conducted between 10:00 AM and 12:00 PM, a late-morning to early-afternoon period during which gas exchange is relatively stable. All measurements were performed under controlled and consistent environmental conditions.

#### 4.1.3. Photosynthesis Pigment Measurements

The content of photosynthetic pigments (Chl a, Chl b, and T Chl) and carotenoids was evaluated. Accordingly, 0.1 g of fresh and powdered leaf tissue was extracted in 2 mL of 96% ethanol. The extract was centrifuged at 13,000 rpm for 15 min, and 1 mL of the supernatant was used for absorbance measurements with a spectrophotometer at wavelengths of 663 nm, 645 nm, and 470 nm (Perkin Elmer Lambda 365 UV-Vis, Shelton, United States), using the formula given below, and the results were expressed as mg g^−1^ FW [38].

Chl a (mg/g FW) = 12.25 A663 − 2.79 A645

Chl b (mg/g FW) = 21.50 A645 − 5.10 A663

T Chl (mg/g FW) = Chl.a + Chl.b

Car (mg/g FW) = 1000 A470 − (1.82 Chl.a) − (85.02 Chl.b)/198

#### 4.1.4. Anthocyanin Measurement

For anthocyanin quantification, 20 mg of fresh leaf samples were ground in liquid nitrogen and homogenized in 2 mL of acidified methanol (1% HCl), followed by incubation at 4 °C for 24 h, and the supernatant was centrifuged at 13,000 rpm for 15 min. Subsequently, all extracted samples were read at a wavelength of 530 nm and 657 nm using a spectrophotometer, as described by Teng et al. (2005), and the results were expressed as Anthocyanin mg g^−1^ FW [39].

#### 4.1.5. Total Soluble Carbohydrate (TSC) Determination

Total soluble carbohydrate content was measured using the phenol-sulfuric acid method [40]. Fresh leaf tissue (0.05 g) was ground in liquid nitrogen, extracted with 5 mL of 95% ethanol, and centrifuged, and the pellet was re-extracted with 5 mL of 70% ethanol. The combined supernatants were centrifuged at 4500 rpm for 15 min. A 100 μL aliquot of the alcoholic extract was mixed with 9 mL freshly prepared anthrone reagent (150 mg anthrone + 10 mL 72% H_2_SO_4_), heated in a water bath for 10 min, and cooled, and the absorbance was measured at 625 nm. The concentration of TSC by drawing calibration curves was expressed as mg g^−1^ FW.

### 4.2. Statistical Analysis

Analysis of variance was performed in a completely randomized design and factorial arrangement using R software (version 4.1.3). The mean comparisons were conducted using Duncan’s multiple range test at the 5% probability level (*p* ≤ 0.05). Statistically significant differences among the treatments were identified accordingly. Pearson’s correlation analysis was applied to determine the relationships between dependent variables, followed by heatmap visualization, PCA-biplot, Euclidean distance, and multi-criteria decision-making using the entropy-weighted TOPSIS method to rank the eight treatment combinations based on 16 physiological traits. These analyses, except for ANOVA and Duncan’s test, were implemented using Python (Google Colab; 3.13.6) with pandas, numpy, seaborn, scikit-learn, and pymcdm libraries to rank the eight treatment combinations based on 16 physiological traits.

## 5. Conclusions

This study demonstrates that the interaction between light spectral quality and VPD plays a decisive role in regulating growth, biomass allocation, pigment biosynthesis, and physiological adaptation during ex vitro acclimatization of micropropagated pistachio plantlets. Red–blue light under low VPD consistently outperformed other treatments, promoting greater leaflet number, stem diameter, root length, leaf area, biomass accumulation, and pigment content. Multivariate analyses confirmed the strong association of red–blue with low VPD with traits underpinning photosynthetic efficiency and structural robustness, while also highlighting the limited effectiveness of blue or white light under high VPD. The findings suggest that integrating optimized spectral composition with favorable evaporative demand can accelerate autotrophic development, reduce transplant shock, and enhance survival in woody plant acclimatization. These insights provide a practical framework for refining controlled-environment protocols in commercial micropropagation systems and for improving the field performance of pistachio rootstocks.

## Data Availability

The original contributions presented in this study are included in the article/Supplementary Material. Further inquiries can be directed to the corresponding authors.

## Supplementary Materials

The following supporting information can be downloaded at https://www.mdpi.com/article/10.3390/plants15030460/s1, Table S1. Comprehensive results of principal component analysis (PCA-biplot) and contribution of traits along the first two principal components (PC1 and PC2) in the principal component analysis (PCA).
