# Supplementary material for: Enhancing Acclimatization of Micropropagated Pistachio Through Optimization of Light Spectrum and Vapor Pressure Deficit
Source: Plants (Basel). 2026 Feb 2;15(3):460. doi: 10.3390/plants15030460 (PMC12899129; doi:10.3390/plants15030460)
Supplement: Supplementary file 1 [file plants-15-00460-s001.zip › plants-4022264-supplementary.pdf]

Supplementary Table of the Article

Enhancing Acclimatization of Micropropagated Pistachio through Optimization of Light Spectrum and Vapor Pressure Deficit

Supplementary Table S1. Comprehensive results of Principal Component Analysis (PCA-biplot), and contribution of traits along the first two principal components (PC1 and PC2) in the principal component analysis (PCA).

Traits	PC1	PC2	Contribution_PC1(%)	Contribution_PC2(%)	Total_Contribution(%)
**Stem L**	0.971844	-0.30099	10.56080408	1.160441544	6.179434868
**Stem D**	0.290375	1.025665	0.942802572	13.47476135	6.783762233
**Shoot L**	0.979814	-0.28675	10.73472711	1.053193433	6.222308208
**Root L**	1.04646	0.165593	12.24471857	0.351230905	6.701340799
**Shoot N**	0.110569	0.998268	0.136699955	12.76453109	6.022344299
**Leaf N**	0.786602	0.537209	6.918521459	3.69654918	5.416808084
**Leaf T**	0.506489	-0.69075	2.868427067	6.111516323	4.379982752
**Leaf FW**	0.958874	0.463797	10.2807832	2.755277626	6.773256942
**Leaf DW**	0.732278	0.75107	5.995914556	7.225560235	6.569034125
**Cl T**	-0.55553	0.867932	3.450747678	9.64899094	6.339656707
**Cl b**	-0.70549	0.797329	5.565309984	8.143016769	6.766740791
**Cl a**	-0.4358	0.97045	2.123637706	12.06304397	6.756247186
**TSC**	1.04646	0.165593	12.24471857	0.351230905	6.701340799
**Car**	-0.4358	0.97045	2.123637706	12.06304397	6.756247186
**Leaf A**	0.996205	0.244605	11.09688005	0.766373062	6.281984337
**Ant**	0.492455	0.808425	2.711669727	8.371238704	5.349510681

Stem L; stem length, Stem D; stem diameter, Shoot L; shoot length, Root L; root length, Shoot N; number of shoots, Leaf N; number of leaves, Leaf DW; leaf dry weight, Leaf FW; leaf fresh weight, Leaf T; leaf temperature, Leaf A; leaf area, Car; carotenoid, TSC; total soluble carbohydrate, Chl a; chlorophyll a, Chl b; chlorophyll b, T Chl; total chlorophyll, Ant; anthocyanin. Note: RB (Red-Blue), R (Red), B (Blue), W (White); Low = low vapor pressure, H = high vapor pressure.
